# Male Nursing Practitioners and Nursing Educators: The Relationship between Childhood Experience, Social Stigma, and Social Bias

**DOI:** 10.3390/ijerph17144959

**Published:** 2020-07-09

**Authors:** Luis Miguel Dos Santos

**Affiliations:** Woosong Language Institute, Woosong University, Daejeon 34514, Korea; luismigueldossantos@yahoo.com

**Keywords:** childhood experience, nursing, nursing development, nursing education, nursing practitioner, nursing school, social bias, Social Cognitive Career Theory, social stigma

## Abstract

The population of nurses and nursing educators is facing significant human resource shortages. One of the pathways to combat this shortage is to recruit male individuals. However, due to social bias and social stigma, the social context may prevent male individuals from joining. There are two purposes of this study. First, this study aims to explore how the childhood experiences of these male nursing practitioners and nursing educators influence their educational decision. Second, from the perspectives of male nursing practitioners and nursing educators, the study aims to explore how the participants describe the relationships between their childhood experiences and lived stories. Based on Interpretative Phenomenological Analysis, the researcher collected data from 10 experienced male nursing practitioners and nursing educators in the United States. The general inductive approach was employed to categorize the themes. The results indicated that early life experiences, positive working experiences, and sense of belonging in the field of nursing always allowed the participants to overcome the social bias and stigma regarding the occupational bias of the nursing profession. The outcomes of this study provide clear recommendations to educators, policymakers, school leaders, and human resource planners to encourage gender social justice and improve their current curriculum for potential nursing professionals.

## 1. Introduction

### 1.1. Introduction and Background

The medical profession is traditionally considered a male-dominated industry and profession. In other words, most of the medical professionals are male individuals due to traditional expectations and social biases. However, although nursing is one of the professions in the field of medical sciences, female individuals make up a large proportion of the nursing profession population. According to a report from the Kaiser Family Foundation [1], as of March 2020, there were 236,772 nursing practitioners in the United States. There were 22,542 nursing professionals who declare themselves as male individuals, while 196,557 nursing professionals declare themselves as female individuals. To illustrate, 83% of female nursing practitioners are serving the communities while only 9.5% of male nursing practitioners are currently working in the profession, respectively [2]. In other words, the gender balance between two genders is vital, which may cause social biases, workplace discrimination, and gendered concerns [3,4].

In fact, it is not uncommon to find different types of social stigma and social bias placed against male individuals joining the female-dominated profession in the field of nursing and social caring [5]. Conservatively, school counsellors, family members, friends, and even general members of the public may prevent their male acquaintances from joining the nursing profession [6]. However, for male nursing professionals who joined as nurses, they may notice that they are in the minority in their working environment.

Although American colleges and universities always increase their numbers of enrolment and develop evening nursing programs and blended learning programs for both traditional-age students and second-career nursing students [7], the numbers of male student enrolments have not significantly increased [8]. According to the Bureau of Labor Statistics, U.S. Department of Labor, the job outlook estimates that there was a 12% increase from 2018 to 2028. Therefore, there will be the potential of nearly one million additional openings in the nursing profession. As for the potential career opportunities for the coming decade, the numbers of male nursing professionals will increase [9].

Although the social stigma and social bias toward male nursing professionals, practitioners, and educators are not unique, only a few studies cared about this serious issue in the medical field and nursing profession. A previous study [10] investigated 15 registered nurses (RNs) from 26 to 43 in age about how gender roles influenced their practices in the nursing profession. The participants indicated that due to their gender, male nurses were arranged to station in certain specializations and departments for more masculine identification, masculine companionship, and reduce the social stigma and biases. Although some indicated that they had no interests in some departments, they could not follow their wills based on managerial decisions (i.e., gender and social stigma as men).

Another study [11] investigated how male nurses described their experiences through a co-cultural lens. Many participants indicated that although their female co-workers expressed welcoming and positive behaviors and feelings, many exercised gender expectations, such as labor works due to social biases and social stigma.

Stress and depression among male nursing professionals due to social stigma and social biases is not uncommon. A previous study [12] indicated that the pervasive sense as a minority in a predominantly female occupation always made male nursing professionals feel negative. Also, many indicated that they had to arrange their understanding and gender roles between the masculinity identity and the nursing role due to their working environment, co-workers’ views, patients’ perspective, and even members of the public. From a historical perspective, the conception of female and male nursing professionals was established, which is attentive to managerial powers and political understanding [13]. Another study [13] also indicated that the gender role would limit their opportunities and developments as nursing is a predominately female profession. These limitations might, however, result in stress and depression among male nursing professionals due to social stigma and social biases.

### 1.2. Purpose of the Study

A recent report indicated that the number of male individuals working on a Bachelor of Nursing degree is slightly over 10%. For postgraduate degrees, such as Master of Science Nursing and Doctoral-Level nursing degrees, these are only made up of 9.9% and 6.8% males, respectively [2]. In order to increase the overall percentage and number of male individuals, it is important to understand the reasons why male individuals decide to enter the field [6]. Particularly, this research study aims to understand how childhood experience and social context influence the career decision of these male nursing professionals. As a result, the researcher listed the following key points.

Firstly, due to the continual shortage of nursing professionals, particularly male nursing practitioners and nursing educators, this research study aims to explore how childhood experiences of these male nursing practitioners and nursing educators influence their educational decision in the first place [14]. For example, what childhood experiences influenced their educational decision (i.e., studying nursing degree and license in the first place).

Second, as mentioned above, social context, social stigma, and social biases are three of the elements that prevent potential male nursing students and nursing potentials from joining the nursing profession [15]. In other words, male nursing professionals face different levels of pressures which prevent their decision-making process. Therefore, from the perspectives of male nursing practitioners and nursing educators, the second purpose aims to explore and understand how the participants describe the relationships and the connections between their childhood experiences and the lived stories about these elements and situations.

### 1.3. Contribution to the Current Literature

Unlike other quantitative research studies which employed a survey and questionnaire, this qualitative research study employed in-depth interviews with lived stories shared for data collection. The result of this research may increase knowledge in three areas regarding the current literature.

First, due to the uniqueness of this research study, the researcher could only recruit small numbers of participants. The detailed requirements are listed in the methodology chapter. Although the research study cannot cover the whole population in the country or the region, the rich, detailed, and unique data, lived stories sharing, personal understanding, and three-decade-long feedback highly satisfy the potential gaps of the literature [16].

Second, the nursing education and health sciences educator’s career decision and career development were not explored by many scholars and researchers, particularly for the male nursing education and health sciences educator who teaches in foreign countries, regardless of school status. Although there are some studies [17] that have indicated some meaningful results, such as financial and personal consideration, most of the nursing school teachers left their positions within their first few years of teaching service. Therefore, the current research study indicated and explored why male nursing practitioners and nursing educators decided to invest their life-long career and career development in the United States.

Third, the results of this research study can provide some managerial insight and recommendations for nursing school administrators and leaders to improve and upgrade professional development planning and managerial strategies for their teachers and school professional staff, particularly in the field of nursing education and human resource management. Therefore, the results from this research study serve as a blueprint and background for appropriate parties to reconsider their managerial planning [18].

## 2. Methodology

This study employed a qualitative methodology [19] to analyze the research study. In the process, the researcher aimed to explore the data information, sharing, and the career decision of why male nursing practitioners and nursing educators decided to invest their life-long career development in this female-dominated profession (i.e., nursing profession) and how male nursing practitioners and nursing educators described their experiences [20]. The qualitative approach allowed the researcher to listen to the participants’ lived stories and explore whether there was a connection.

### 2.1. The Application of the Interpretative Phenomenological Analysis

Based on the qualitative research methodology, this study followed Interpretative Phenomenological Analysis (IPA) as a tool to investigate the problem. The perspectives of IPA tend to seek the understanding, sense-making process, feedback, inner world, and the concepts of the individuals and groups. Unlike other qualitative research methods and tools, IPA has the characteristics to seek the in-depth and inner world of individuals and how the individuals would describe their lived stories, life experiences, and sense-making process. Therefore, the data information of the participants must be rich, meaningful, and intensive. Also, the researcher always tried to seek the social and inner world of the individuals. The researchers should always enter and place themselves into the individuals’ situations and events in order to gain a full and holistic picture of their background and experiences.

Based on the guidelines of the IPA methodology, scholars advocated that studies should not employ more than 10 participants for each study in order to ensure in-depth and meaningful data information. In fact, IPA studies always focus on in-depth, meaningful, and intensive data information. Therefore, studies with more than 10 participants may not satisfy these requirements.

### 2.2. Participants and Recruitment

The researcher set up some essential criteria for recruitment. In other words, the potential participants needed to meet all the following elements:

1. Male individuals;
2. Currently teaching or providing educational services in a nursing school setting;
3. Participant must be a registered nurse;
4. Participant has worked in the field of nursing for at least 20 years;
5. Participant plans to continue his teaching position in the field of nursing education.

After the researcher set up the criteria of the participant, the researcher started the recruiting procedure and five steps were established. First, to recruit such unique participants in the United States, the researcher sent out an email invitation with the five-point criteria to different nursing schools. Second, two months after the researcher sent out the email invitation, most of the respondents indicated that no nursing education and health sciences educators met the criteria. Most of the emails indicated that nursing education and health sciences educators usually left their school within the first few years of their teaching service. Third, about three months after the email had been sent, several nursing schools indicated that they had one nursing education and health sciences educator that met the criteria and were willing to participate in this research study. Fourth, once the school administrators agreed to forward the contact details of the potential participant to the researcher, the researcher contacted the potential participant immediately with the research protocol and interview questions. Fifth, the participant agreed to meet the researcher in person for the in-depth interview sessions. The following part explains the in-depth interview procedures. It is worth noting that all 10 participants were White/Caucasian people who were born and raised in the United States. The following Table 1 lists the demography of the participants.

### 2.3. Data Collection

According to Seidman [21,22], participants usually do not share lived stories and sensitive information to strangers—in this case, the researcher. To build up an established relationship, the researcher needed to conduct two private interview sections to gather useful interview information [23]. However, due to the location and COVID-19 lockdown during early 2020, the researcher could only host the interview sessions online via Social Media Application live chat (i.e., WhatsApp). Lived stories, experience, and interactions within the region were rich and colorful and the researcher decided to employ open-ended interview questions [24] as semi-structured and structured questions may potentially limit some lived segments [25]. For the general direction for each of the interview sessions, the researcher aimed to capture data for three elements of life, including

- How would childhood experiences influence your sense-making process about nursing education, career decision, and development;
- How would childhood experiences influence your sense-making process about switching from frontline nursing practitioners to nursing educators;
- How would the participants describe their experience as male nursing practitioners and nursing educators and the relationship among social context, social stigma, and social bias?

It is worth noting that each of the interview sections lasted 68–111 min. The researcher used a digital recorder (within the computer system) to record all the voice interviews. After the completion of two interview sections, all the voice records were transcribed into written transcripts. In addition to the lived stories and sharing from the participants, the researcher also encouraged the participants to show their valued items and memories to the researcher for reporting. A general inductive approach was employed to analyze the interview materials [26].

### 2.4. Data Analysis

Themes and patterns that grouped during the interview sessions were independently categorized. A general inductive approach [26] was employed to analyze this research study. The general inductive approach allowed the researcher to understand the interview transcripts and feedback from the participant. The study employed IPA as a research tool for the investigation. After the data collection procedure, the general inductive approach served as a tool to categorize the data information into themes, groups, and categories.

In fact, the general inductive approach is a useful tool to narrow down large-size data information into meaningful groups and categories. Like many qualitative research studies [5,14,27,28,29,30,31,32,33,34,35,36,37,38,39,40,41,42], the researcher followed an inductive approach and direction to understand and read the data. Unlike case study, narrative approach, and grounded theory studies, the researcher could not follow any data analysis procedures of these methodologies due to the nature of the focuses and aims. Therefore, the general inductive approach served as an appropriate tool for the data analysis procedure in this study. The following part will explain the procedure and application.

First, the researcher followed the general inductive approach to narrow the massive interview transcripts into first-level themes by using an open-coding strategy [19] from the ideas of the grounded theory approach. Many researchers and scholars advocate that researchers should read through all the transcripts. As for the first-level themes, the researcher was able to group 20 themes and 18 subthemes based on the large amount of information from the transcripts.

**Figure 1:**
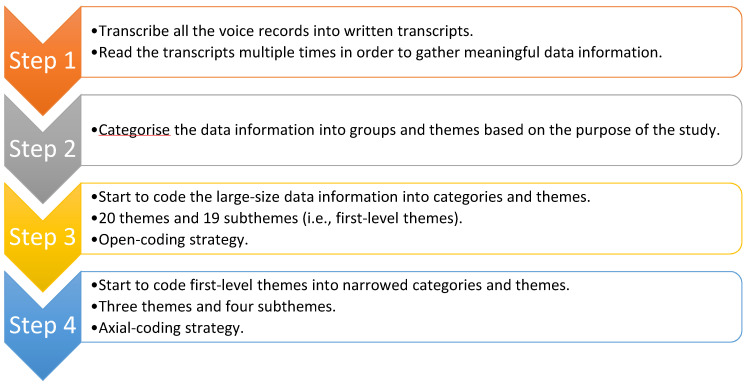
The procedure of the data analysis.

Second, the researcher further followed the recommendation from the general inductive approach. Therefore, the axial-coding strategy was used to further reduce the data into second-level themes. Creswell [25] and Merriam [19] advocate that the axial-coding strategy allows the researcher to group all central themes and subthemes into narrowed categories and groups. As a result, three themes and four subthemes were categorized for reporting. Figure 1 outlined the overall procedure of the data analysis process.

### 2.5. Human Subject Protection and Ethical Consideration

Due to the limited personnel in the field of nursing practice and nursing education, particularly for male professionals in the field, confidentiality was the greatest concern of this research study. The researcher has made every effort to protect the participants’ personal information. Therefore, the researcher arranged for each participant to have a pseudonym. Due to the content form and agreement, the researcher needed to mask the real name(s), age, related colleges and universities, and employment location of the participants.

All signed and unsigned content forms and agreements, personal contacts, audio recordings, transcripts, computers, and related materials were locked in a password-protected cabinet. Only the researcher has the rights to read the information. After the data analysis, the researcher destroyed and deleted the related materials due to reasons of privacy. All subjects gave their informed consent for inclusion before they participated in the study. The study was conducted in accordance with the Declaration of Helsinki and the protocol was approved by the Ethics Committee of the Woosong University.

## 3. Results

During the interview sessions, the participants answered the same general open-ended questions about their lived experiences for career decisions related to childhood experiences and the social stigma and social biases based on the current American social context. To build up a solid relationship with the participants, the researcher should travel to the participants’ office. However, due to the COVID-19 lockdown, face-to-face interview sessions were not encouraged. Therefore, both agreed on online interview sessions as an alternative. Although the research study only captured the lived stories from 10 participants (*N* = 10), the data information and lived stories sharing were very rich and detailed, which may cover the meaningful background of the study.

The researcher responded positively and asked them to share their stories. Many shared their thoughts on how to motivate pre-service, junior, mid-level, and senior-level registered nurses, nursing practitioners, and nursing educators and how including teachers’ professional identity in a professional development program for teachers with various cultural and learning backgrounds and perspectives could be beneficial. Table 2 outlines the themes and subthemes of this study. It is worth noting that the following themes and subthemes are a result of the second-level themes from the axial-coding strategy.

### 3.1. Early Life in their Hometown: Sense of Nursing Education, Career Decision, and Development

For the first interview session(s), the protocol focused on the childhood experiences of the participants. Many began the conversation by sharing their lived stories and explaining why as a young man they decided to study for a bachelor’s in nursing at university.

It is worth noting that all 10 participants were inspired by their family members. For example, a participant (Participant #3) said that his mother was a primary school teacher who taught in his hometown for more than 30 years until she retired at the age of 65. In the early 1990s, Participant #3′s mother decided to become a teacher at a community center for Asian immigrants near the border between the United States and Canada. Participant #3′s maternal grandmother was born in Canada. She observed a large number of Asian immigrants coming to Canada due to the racial discrimination they experienced. As there were no teachers or care providers for these Asian immigrants, Participant #3′s grandmother decided to assist. If Participant#3′s grandmother was not available to teach these immigrant learners, his mother took on the role of teaching them, particularly in basic English lessons and health promotion. Participant #3 was proud of his mother’s service to poor and hopeless immigrants in his hometown. Accordingly, learners in the region who had been educated by his grandmother always called his mother and grandmother “teachers”, *sensei* in Japanese, *laoshi* in Chinese Mandarin, or *sinsaang* in Chinese Cantonese. Participant#3 commented on the voluntary nature of the teaching service his grandmother and his mother provided.

#### 3.1.1. Volunteering Service

Due to their early childhood experiences with family members and closed community networks during the past decades, many participants established their open-mindedness through relationships with classmates, friends, and mates with different cultural backgrounds, family backgrounds, languages, and even skin colors. Although the United States is noted as one of the most diverse countries, many of the residents in the country might not have the chance to interact with different people during their childhood. However, these childhood experiences highly increased many participants’ sense of globalization and internationalization. For example, Participant#1 also participated in many volunteering services with his family members during his childhood, saying:

*If they had charged these poor children and adult learners for their services, these people might not have come to learn as they did not have enough money to buy basic food. Some of them needed to send all of their salaries back to their hometown. So, my grandmother and mother never charged for the teaching service they provided*.

(Participant #1)

Another participant also spoke to his volunteering services and experiences which influenced his sense of nursing and career decision of entering the nursing profession. For example, Participant #4 said that his family always visited the elderly housing for volunteering services during his childhood. Such experiences and lived stories highly influenced his career decision (i.e., joining the nursing school after secondary school graduation), as he said:

*Serving the senior residents in our country was very meaningful…we went to the senior housing every week…we helped all senior citizens…my mom told me that we will get old in the future, we needed to help people who need us…we continued with this volunteering services until the last minutes of my mom…that’s why I decided to continue my education in nursing…*.

(Participant #4)

Besides doing volunteering works in elderly housing, several participants experienced volunteering services during their childhood in orphanages. Such experiences in the orphanages might have highly influenced their career decision and sense-making process (i.e., joining the nursing school after secondary school graduation). For example, the researcher captured two meaningful conversations, one said:

*…doing the volunteering works in the orphanages…were very great…my parents told me that we have to take care of hopeless children…children are the future of our community and country…although they do not have parents, we can send love and caring to them…some children were fostered by good families…this is a non-stopped process…I can see the progression. I grew, and they grew as well…so that’s why I start my nursing degree…*.

(Participant #8)

Another participant also advocated that volunteering services and experiences with children always increased his sense of belonging in the field of nursing and social caring. The participant spoke to his childhood experience with his parents in the Asian American community center, sharing:

*I always enjoyed my time with my mother and sisters in the Asian American community center near my school…we did a lot of volunteering services…we made friends…we merged other new immigrants into our community…I remembered when some of my peers harmed themselves…we helped each other…I learnt the basic healing skills from the community nurses…and now, I will transfer these skills to the youths in our nation…*.

(Participant #6)

In short, based on some previous studies [18,43,44,45], childhood experiences, family experiences, and background stories from the participants’ hometown always influenced their sense of belonging, sense-making process, and their decision-making process. In this case, many decided to become nursing professionals due to their meaningful childhood experiences and background stories. Such behaviors and decisions highly reflect the previous studies and how previous researchers have explained individuals’ behavioral sciences.

#### 3.1.2. Modelling as Teachers and Educators

In addition to some volunteering service with family members, many participants also learnt some of the teaching and learning models and strategies from their mothers and grandmothers [46,47]. Previous studies indicated that parental influences might impact individuals’ behaviours. Such strategies and experiences also increased their ideas about immigrants, English language learners, and how to interact with English language learners in their living community. Many continued to share lived stories about their family members. For example, Participant #5 shared that after his mother married his father, she continued to volunteer for nearly 15 years until he was 10 years old, saying:

*During my early childhood, I always went to the community center or church with my mother to observe the volunteering service. The teaching I observed was beneficial and interesting…from new immigrated learners to experienced immigrants, was engaged in learning...both English and health education…I could see the growth of others as well as my own personal growth. I also met one of my best friends at the community center during my childhood…without these experiences, I could not select my university major in nursing or social caring services*.

(Participant #5)

Besides Participant #5, several others also shared their lived stories about volunteering and teaching at the community centers and churches. For example, Participant #10′s mother was a registered nurse in the field of health promotion. During the weekends, after religious services, his family always went to the community centers for health promotion for new immigrants from the East Asian region. It is worth noting that his childhood experience highly influenced his university selection and further career decision as a current nursing educator. He said:

*Besides basic volunteering services, my mother…taught lessons in sexual promotion, sexual health, condom use, and underaged sexual intercourses etc… My mother and my family members also benefited from this critical teaching service…Every Lunar New Year, more than 100 Chinese current or former learners came to our farm…[to] bring…Chinese dumplings, sweet soups, and pocket money for my brothers and sisters. I extremely enjoyed this meaningful experiences and services to my community members. Therefore, I want to follow my mother’s step…I want to become a nursing educator*.

(Participant #10)

In short, it is worth noting that from this conversation, many participants’ family members started to teach health and sexual knowledge to immigrants with different cultural backgrounds and expectations. Although sexual issues and topics are taboo in the East Asian cultural background, many participants’ family members continued to teach as this is vital knowledge for all people, including teenagers [48,49]. Therefore, many gained the teaching knowledge and cultural background of teaching, learning, and education during their childhood.

#### 3.1.3. Childhood Peers’ Influence

Due to the active volunteering services with many multicultural community members, friends, and peers in their community, many were able to join some of the cultural holidays and ceremonies during their childhood. Such experiences, according to many previous studies about cultural influences [50,51,52], also increased their interests in being nurses and nursing educators for their career development [53]. First, many explained that the Asian–American cultural combination in the United States allowed them to understand that Western cultures or Caucasian racial groups were only one part of American society. In addition, their intercultural and multicultural experiences about eating habits increased their interest in health promotion and cultural health promotion. To illustrate, the researcher captured a lived story from Participant #6 about how his peer’s Asian cultural practices influenced his sense-making process of being a nurse and nursing educator in the future, as he said:

*During my early childhood, I never left California…The only foreign information such as health knowledge and eating habit, I received was from the Asian immigrant learners…My best friend, James was born in China and moved to California when he was about five years old. He always eats Chinese food and practiced Chinese customs. During the Lunar New Year…I have learnt the eating habit, food sciences, nutrition, and balancing from Chinese people…This unique experience…always influence my career decision…that’s why I love to do health promotion and eating habit promotion at the hospital…*.

(Participant #6)

Besides the Chinese New Year Celebrations with peers in the community, more than half also explained that they always celebrated Japanese New Year and western New Year with Japanese friends and peers during their childhood. During that time, these participants learnt about how to handle health behaviors from Japanese friends and peers during the New Year Celebration and further celebrations. Such childhood experiences also increased their interests in becoming nurses and nursing educators. For example, one said:

*The Japanese New Year is on the same day as the Western New Year…Japanese learners came to our farm for a celebration after worshipping at the Japanese shrine near the edge of the seashore…My Japanese friends and their family always asked me to swim every morning in order to train up our body…They told me that early morning exercise…is…important elements in their lifespan…I was very fortunate to have experienced some colorful East Asian cultures during my early childhood… this experience increased my interest of sport education…further it increased my interests of helping East Asian citizens’ health and related health promotion…*.

(Participant #7)

In fact, the eating habits of East Asian American people and local American people is totally different due to cultural differences. Some previous studies indicated that international personnel might have cultural conflicts in hosted countries due to misunderstanding and cultural differences [46,54,55,56]. Therefore, when western teachers instruct Asian people’s eating habits, the cultural differences and misunderstanding may increase conflicts between the groups. As explained by several participants, such cultural misunderstanding and challenges may reduce the overall performance of health promotion (e.g., if health promotors and nurses do not understand the cultural differences).

In short, although these lived stories during participants’ childhood may not be important to share and experience in their life, such experiences increased their interests and passions to become nurses and nursing educators as their career decision and development. These pieces of lived stories also indicated that many participants’ career decision and career development about becoming nurses and nursing educator were influenced by their unique childhood experience [57,58,59].

### 3.2. My Senses of Caring from My Childhood Experiences: Working Experiences

Here, concerns from the perspectives of male nursing practitioners and nursing educators are discussed. The second purpose aims to explore and understand how the participants describe the relationships and the connections between their childhood experiences and the lived stories about these elements and situations, particularly in the areas of social context, social stigma, and social biases.

#### Positive Working Environment: Overcome the Social Stigma and Social Bias

Although all shared concerns about social stigma and social biases (e.g., being male nurses), most co-workers (i.e., doctors, nurses, therapists, administrators, and staff) shared positive expressions and ideas. Such positive feedback and the first career opportunity in the hospitals highly influenced their career decision and career development due to their childhood experiences and interests [60]. First, four indicated that they decided to enter the health promotion section for immigrants and minorities due to their childhood experiences after they gained Registered Nurse licensure. For example, a participant shared his first working experience in a rural community station for immigrants and racial minorities, saying:

*…I started my career as a bilingual family health nurse and health promotor for some lovely immigrants in a rural community in California…I really think my childhood experiences…changed me a lot…I really want to serve the Asian American population because my parents always asked us to do volunteering services during my childhood…So after I gained my RN status, I joined the Asian American community immediately…*.

(Participant #9)

Not only Participant #9, but several others also shared a similar lived story (i.e., relationships between their career decision and childhood experiences). Such childhood experiences highly influenced their career directions (i.e., entering the areas related to their childhood experiences). For example, the researcher captured another lived story, with one participant saying:

*I first completed my placement and internship at an Asian community clinic…it is because of my childhood experience at the Asian community center…I worked with my parents for sexual health promotion and eating habit promotion...After I completed my placement and internship, I decided to continue…to work with the Asian American community…because my childhood experience absolutely changed my views and understanding…*.

(Participant #10)

The researcher further asked about how they would describe their understanding and situation between their gender and social context, social stigma, and social biases [37,61,62]. All expressed that they faced social stigma and social bias due to their gender and negative feedback from the general public. However, many overcame such difficulties as many have high levels of self-efficacy. For example, one shared how his marketing and communication skills helped the rural community district for the fund-raising promotion, as he said:

*I then continued with this career path as a nurse…during the early stage of my nursing career in the bilingual clinics…some parents and patients did not understand my role as a man…I could see the social stigma and bias of course…but due to my childhood experience about…dealing with bilingual residents…many accepted my role because of my passion…afterward, I could use my bilingual skill…to do some fund-raising and health promotion activities…yes, there are bias, but we can overcome…*.

(Participant #2)

Indeed, social stigma and social bias has always existed in our community, particularly for male nursing professionals. However, many participants overcame these elements and problems with high levels of self-efficacy and positive childhood experiences from their previous lived stories and experiences [37,61,62]. The researcher further captured how this participant managed his mind and overcame some social bias and discrimination from his working environment:

*…not much support or assistance was available for some rural communities, orphanages, and senior housings…during my first four years of services in the rural communities, some residents and parents felt negative because of my gender…but I worked hard to gain the credits from the local residents…I continued my childhood mind…I continued to serve as a volunteer in the senior housing and community centers…eventually, I gained the trusts from the public members in the county…*.

(Participant #4)

In short, all expressed that their gender did not cause any social stigma and social bias from their professional co-workers in the medical profession [37,61,62]. Although many explained that social stigma and social bias existed, they overcame it with passions and hard-work due to their high levels of self-efficacy and positive childhood experiences.

### 3.3. Upgrading my Childhood Experiences and Professional Working Experiences to Nursing Education

Like many other registered nurses and nursing practitioners who planned to work as frontline professionals as their life-long career development, all participants in this study earned their doctoral degree program after years of work experience in the medical profession [57,63]. Surprisingly, all participants had experience in the section of health promotion, school nursing, health administration, or minorities’ health. It is worth noting that these experiences and childhood experiences highly influenced their career switching decision (e.g., from frontline nursing profession to nursing education). For example, one said:

*I used to work in a school as a school nurse for five years as I like to work with students, youths and young children…I learnt how to promote healthy life and well-being at school…not only for our students, but also teachers, staff, and parents in the school district…because of these meaningful experiences from my experience…I decided to upgrade my childhood experiences and professional working experiences to nursing education at the college level…*.

(Participant #8)

All participants advocated that their childhood experiences and working experiences always influenced their career decision to pursue nursing education [3,18,44,45]. For example, Participant #3 advocated that he grew up in a low-income district, worked at the low-income district, and continued to serve the low-income district for the rest of his life. Due to his passion and intention to work for low-income families and community members, he wanted to transfer his knowledge and skills to low-income and college students in a similar community, saying:

*…In rural midwestern United States…where I spent my childhood…there are community colleges, colleges and universities around…after I completed my bachelor’s degree in the state’s capital city, I came back to the rural community for low-income families…a significant number of students were forced to go to the workforce…not [exactly] forced…but not many of them had the opportunities…since there were no vocational and technical schools during that period…so when a college started their nursing program around, I have my intention to join…the teaching profession for my field in nursing…*.

(Participant #3)

It is worth noting that childhood experiences always influenced these participants’ career decisions (i.e., combining childhood experience with working experience in nursing education). In this case, the childhood volunteering experience for the East Asian community members served as a key for upgrading (i.e., from frontline to nursing education) [57,63,64]. A similar sentiment was captured from another participant, who said:

*For many East Asian community in the west coast, most of the students enjoyed school life as they understood that education should not be taken for granted. Many from foreign families [immigrated families] might not be able to afford school, afterschool program, and college education. Therefore, they showed respect…including [towards] teachers. Parents, particularly those from East Asian families, also respected our profession. I heard the words teacher and laoshi in Chinese Mandarin being used instead of my first name…It is this respect…that may change the decisions of teachers…*.

(Participant #7)

In conclusion, based on the sharing from the participants, many advocated that their childhood experiences, lived stories, and background stories highly influenced their sense of belonging, sense-making process, and the decision-making process in the field of nursing professionals and nursing educators. Based on the theoretical framework and the previous studies about childhood and lived experiences, this study indicated a clear connection between these elements.

## 4. Discussions

The current findings indicate that although many faced social stigma and social bias due to their gender roles in the nursing profession [6,40], many overcame the discrimination, social stigma, and social bias due to their strong sense of belonging from their childhood experiences and personal goals. First, many of the participants indicated that their sense of belonging (i.e., being a male nursing professional) was always established and firmed due to their positive early life experiences in their hometown [65]. Previous studies [40,43,66,67,68,69] indicated that individuals often selected their career developments due to their positive early childhood experiences, parental influences, and personal goals. In this case, many participants had joined and served the volunteering services with bilingual and multicultural individuals during their childhood. These experiences, therefore, served as their personal goals and motivations [66,67,68,69,70,71] for joining (i.e., the nursing professionals).

Second, although many indicated that their gender (i.e., being a male nursing professional) might negatively impact their gender identity and gender role as nursing is a predominately female profession due to the social stigma and social bias in communities, many had overcome these negative feelings and thoughts. More importantly, one of the significant findings relied on early childhood experiences from their friends, classmates, parents, and grandparents. For example, like Participant #8 shared, “immigrants” children are the loved ones of others…we have to provide care if we can”. Another participant (Participant #7) also echoed and said, “Japanese American people are American people…we are not going to categorize them…they are American, and they are people”. According to previous studies [66,67,68,69,70,72], this motivation from previous experiences [40] usually directed people’s career perspectives and motivations due to a sense of belonging and positive experiences from lived stories. The current study, therefore, reflected the findings of previous studies and explored the additional sharing and lived stories from this group of male nursing professionals in the United States [6,11].

Third, the findings also discovered that personal goals highly relied on their career perspectives [66,67,68,69,70,72]. Previous studies [5,43,57] indicated that individuals could be influenced by financial consideration, environmental and external factors, personal goals, and educational achievements. Based on the findings of this study, many participants were highly influenced by their personal goals and their childhood experiences [66,67,68,69,70,72]. In fact, the nursing profession and nursing education, as one of the non-profit organizations and developments, individuals usually believed that personal goals are their main purposes for career developments. In this case, regarding male nursing professionals, although many experienced social stigma and social bias due to their gender role, they continued to work and overcome the difficulties as they have a strong sense of belonging and positive childhood experiences. The positive sense of belonging and childhood experiences, therefore, served as their motivation [66,67,68,69,70,72].

## 5. Limitations and Future Research Directions

Each study has its own limitations. For this study, the researcher concluded three limitations for future research directions. First, the number of participants were limited. The current study only recruited 10 experienced participants as the study focused on a topic with a limited population. Although the researcher could invite female participants to join the study, the aims of the study would change as gender-oriented issues would be the key points. Therefore, the focus of this study was unique. The number of the participants could reflect the overall performance and the picture in the current nursing field. Future research studies may expand the study and re-conduct the study again as people may share additional stories and life experiences decades later.

Second, the human resources shortage is an international problem, particularly for the workforce of male nursing professionals. Therefore, future research may expand the horizon to other countries and regions with a similar background and problem. For example, many participants indicated that social and cultural context and the situation might influence their sense of belonging.

Third, as this is a qualitative research study that mainly focused on a group of male nursing professionals in the United States, future research may employ quantitative research tools, such as survey and questionnaire, in order to gather wider data information from different parts of the globe.

## 6. Conclusions

Based on childhood experiences, the study shows how a study and the sharing of lived stories can be used to understand the sense of belonging, sense-making process, decision-making process, and understanding as nursing professionals and nursing educators in a particular country and region—in this case, the United States and male individuals. Further research is required in other regions and locations of the world. As the research approach for this research was conducted successfully, future researchers could follow the same methodology and data collection procedures, with appropriate revisions, for similar research studies. For example, researchers could invite participants to participate in three interview sessions in order to build a relationship and capture life-long lived stories and experiences within a reasonable time frame. It is worth noting that researchers should employ open-ended interview questions based on the actual situation during interview sessions [24]. This raises the issue of whether the narrative content of amazing lived stories and experiences could be developed in further directions.

## Figures and Tables

**Table 1:** Demography.

Name	Position	Age	Highest Degree
Participant#1	Nursing Practitioner Nursing Educator	45+	Doctor of Nursing Practice
Participant#2	Nursing Practitioner Nursing Educator	45+	Doctor of Nursing Practice
Participant#3	Nursing Practitioner Nursing Educator	50+	Doctor of Nursing Practice
Participant#4	Nursing Practitioner Nursing Educator	50+	PhD Nursing
Participant#5	Nursing Practitioner Nursing Educator	50+	PhD Nursing
Participant#6	Nursing Practitioner Nursing Educator	50+	Doctor of Nursing Sciences
Participant#7	Nursing Educator	50+	Doctor of Public Health
Participant#8	Nursing Educator	50+	Doctor of Public Health
Participant#9	Nursing Educator	50+	PhD Public Health
Participant#10	Nursing Educator	50+	PhD Public Health

**Table 2:** Themes and subthemes.

Themes and Subthemes		
3.1		**Early Life in their Hometown: Sense of Nursing Education, Career Decision, and Development**
	3.1.1	Volunteering Service
	3.1.2	Modelling as Teachers and Educators
	3.1.3	Childhood Peers’ Influence
3.2		**My Senses of Caring from My Childhood Experiences: Working Experiences**
	3.2.1	Positive Working Environment: Overcome the Social Stigma and Social Bias
3.3		**Upgrading my Childhood Experiences and Professional Working Experiences to Nursing Education**
